# Parents of Premature Infants Value Early Skin‐to‐Skin Care: A 10‐Year Survey in France

**DOI:** 10.1111/apa.70387

**Published:** 2025-11-26

**Authors:** Odile Dicky, Pierre Kuhn, Jacques Sizun, Madeleine Akrich, Charlotte Bouvard, Charlotte Tscherning, Laurence Caeymaex

**Affiliations:** ^1^ Neonatal Intensive Care Unit University Hospital Toulouse France; ^2^ UMR 1027 INSERM Paul Sabatier University Toulouse France; ^3^ Neonatal Intensive Care Unit University Hospital Strasbourg France; ^4^ Cognitive and Adaptative Neuroscience Laboratory CNRS Strasbourg France; ^5^ Neonatal Research Unit, Department of Women's and Children's Health Karolinska Institute Stockholm Sweden; ^6^ Collectif inter‐associatif autour de la naissance Paris France; ^7^ Association SOS Prema Neuilly‐sur‐Seine France; ^8^ Neonatal Intensive Care Unit Oslo University Hospital Oslo Norway; ^9^ Institute of Clinical Medicine University of Oslo Oslo Norway; ^10^ Institut Toulousain des Maladies Infectieuses et Inflammatoires (Infinity) Toulouse University, INSERM, CNRS, UPS Toulouse France; ^11^ Neonatal Intensive Care Unit Centre Hospitalier Intercommunal de Créteil Créteil France; ^12^ Faculty of Health Paris Est Créteil University Créteil France; ^13^ Centre d'Etudes Discours Images Textes Ecrits Communication (CEDITEC) Paris Est Créteil University Créteil France

**Keywords:** experience, Kangaroo care, neonatal intensive care unit, parental involvement, parenting, patient‐ and family‐centred care, premature infant, skin‐to‐skin care

## Abstract

**Aim:**

Skin‐to‐skin contact (SSC) is a key aspect of infant‐ and family‐centred developmental care and should be initiated as early as possible after birth. This study aimed to assess the effects of early SSC, within the first 3 days of life, compared to late SSC, from Day 4 onwards, in very preterm infants. It also explored parents' experiences of SSC.

**Method:**

Data were collected over 10 years, from 2014 to 2024, via an online parental survey created by parent associations and the French National Neonatal Society. We analysed only responses from parents of infants born before 32 weeks' gestation.

**Results:**

A total of 2212 responses were analysed. Findings revealed that 98% of parents who began SSC early believed it occurred at the right time, while 30% of those who started later felt it was too late. Parents who practised early SSC were more likely to report comfortable SSC during the hospital stay, with an adjusted odds ratio of 1.64 (95% confidence interval: 1.14–2.37). Key factors contributing to a positive SSC experience included support from nursing staff, a secure setting, privacy and a comfortable chair.

**Conclusion:**

Early SSC was highly valued by parents of very preterm infants.

## Summary

- Skin‐to‐skin contact soon after birth is advised for preterm infants, but parents' views on it remain under‐researched.
- This 10‐year survey, from 2014 to 2024, found that 98% of parents who practised early SSC felt the timing was right and boosted parental confidence for SSC.
- These findings highlight the need for healthcare professionals to encourage early SSC to better meet parental expectations and infants' developmental needs.

**Abbreviation**
SSC: skin‐to‐skin contact

## 1 Background

During skin‐to‐skin contact (SSC), the baby is positioned naked on the parent's bare chest, wearing only a nappy [1, 2]. SSC has been shown to have many short and long‐term benefits for preterm infants including improving physiological stability, growth, immunity, neurodevelopmental outcome and social behaviour until early adulthood [3, 4, 5, 6, 7]. SSC also increases breastfeeding rates even beyond the neonatal period and decreases readmissions. Furthermore, SSC in preterm infants promotes sleep cycling, and consequently accelerates brain maturation. There seems to be an even greater positive impact with prolonged SSC [8, 9]. There are also many benefits for the parents, such as facilitated parental sensitisation to their infant's needs and cues, improved parental empowerment and self‐efficacy, and reduced stress [10, 11]. SSC also provides the opportunity for early bonding between the infant and a safe and supportive parent figure [12]. This is particularly important for very preterm infants born before 32 weeks' gestation, considering the recognised challenges in establishing future secure attachment. In this vulnerable population at increased risk of neurodevelopmental impairments, healthcare professionals should encourage and help parents to practise SSC as soon as possible after birth [13, 14]. The World Health Organization recommends early and prolonged SSC, or intermittent Kangaroo Mother Care for very preterm infants [15]. Research has been focused on barriers faced by healthcare professionals concerning the practice of SSC, but few papers have addressed the feelings of parents about the timing of the first SSC [16, 17]. Describing parents' feelings during SSC, identifying the causes of stressful SSC and understanding parents' views on how to improve the SSC experience are important to more widely implement early SSC for preterm infants and their parents. Since 2014, the Group for Reflection and Evaluation of the Environment of Newborns, part of the French Society of Neonatology, has worked on various environmental issues to improve care for hospitalised newborn infants [18]. Written recommendations to promote SSC were made available online and published in 2018 by the French Society of Neonatology [2].

Our primary objective was to analyse how parents felt about their SSC experience during their very preterm infant's hospital stay, and to identify factors associated with early SSC. We defined early SSC as starting within the first 3 days of life. Our secondary objective was to compare the experiences of very preterm infants' parents who practised early SSC with those who practised SSC from Day 4 onwards.

## 2 Patients and Methods

Data for this study were derived from responses to an online survey launched in 2014 by the French Neonatal Society [19], in collaboration with two French national non‐profit, state‐approved user associations: *Collectif interassociatif autour de la naissance* [20] and *SOS Prema* [21]. These associations are dedicated to improving baby‐ and family‐centred care in France, both at birth and during neonatal hospital stays. *Collectif interassociatif autour de la naissance* focuses on promoting parental empowerment and respectful, personalised care centred on the mother, baby and family. *SOS Prema* supports parents of premature infants and hospitalised neonates, and advocates for their rights. The survey aimed to explore the practices of neonatal units across France and to ascertain parents' experiences with these services. It was designed to remain accessible to new parents over time, facilitating the collection of longitudinal data. This approach allowed the study to draw on experiences from a large number of units and parents over an extended period. The survey was promoted through social media, parent associations, healthcare providers and perinatal networks. The questionnaire comprised 222 items. Completing the full questionnaire until the last question would take approximately 30 min, and around 20% of respondents left the survey incomplete. Questions regarding SSC began at item 85.

### 2.1 Participants

For this study, we analysed responses from parents of infants born before 32 weeks' gestation who completed the survey over a 10‐year period, from 2014 to 2024. Both parents were eligible to participate.

### 2.2 Outcome Measures

Data for this study included responses on parent and infant characteristics and data derived from specific questions about skin‐to‐skin care. Based on responses to this question, parents were categorised into one of two groups according to the timing of the first SSC: early SSC before Day 4 of life, and late SSC from Day 4 onwards. A multiple‐choice question assessed the timing of the first skin‐to‐skin contact, with possible responses including: within 3 days of life, from Day 3 to 7, from 1 to 2 weeks, from 2 to 3 weeks, or more than 3 weeks. Other questions explored whether parents thought the first skin‐to‐skin contact occurred too early, at the right time or too late. They also examined whether parents felt comfortable practising skin‐to‐skin contact during their baby's hospital stay. Two open‐ended questions enabled parents to describe their experiences in their own words. These asked why they felt uncomfortable during SSC and what support they needed. They also explored what parents considered essential for feeling comfortable during SSC. Lastly, one question was about the name of the neonatal unit. This allowed us to document any limitations in terms of the validity and generalisation of the results [22].

### 2.3 Statistical Analyses

Parental characteristics, along with those of the pregnancy and preterm infants at birth were compared between the two groups using bivariate analyses. Next, we analysed how parents felt about and experienced SSC throughout their infant's hospital stay. Multivariable regression analyses were then conducted to assess how the timing of the first SSC affected parental feelings, after identifying potential confounders. Confounding factors were selected by identifying common causes that could be associated with the timing of the first SSC and parental feelings. These factors included the parents' social, professional and marital status, characteristics of the pregnancy, the baby's characteristics and the type of neonatal unit at birth. A stepwise analysis was performed using a Poisson regression model, with the late SSC group serving as the reference group. Sensitivity analyses were subsequently conducted to estimate the effect that the timing of the first SSC had on parental feelings, checking for potential intermediate variables [23]. These are variables that could be mistakenly considered confounding factors and lie on a causal pathway between the timing of the first SSC and parents' feelings. All statistical analyses were performed using Stata Statistical Software 11.0 (Stata Corporation, College Station, TX, USA). Written responses to the open‐ended questions were analysed by two investigators using discourse analysis [22]. The researchers were unaware of the clinical and demographic characteristics of the patients and their parents. The two researchers conducted an initial analysis of the entire corpus to identify themes and subthemes, which they then discussed to reach a consensus. Each researcher then coded the themes and subthemes emerging from the data independently, to optimise the validity of the results. Special attention was paid to unusual comments or contradictory data. Then, the researchers compared their classifications, reassessing the data until a consensus was reached in case of discrepancies.

### 2.4 Ethics

The French National Data Protection Authority approved this questionnaire. The Ethics Committee of Strasbourg University approved the study protocol in 2016, under approval number 2015‐45. Informed consent was obtained from all participants included in the study. The survey was individual and anonymous. Parents stated whether they agreed or refused to have their comments published in a research paper.

## 3 Results

Between February 2014 and March 2024, a total of 2212 parents of very preterm infants responded to questions regarding their feelings during SSC, the majority of whom were mothers (96%). Table 1 presents the characteristics of the parents and infants, comparing them according to the time of the first SSC. Only 987 parents (45%) reported engaging in SSC in the first 3 days of life, while 1225 parents (55%) started from Day 4 onwards. Compared with the late SSC group, parents in the early SSC group were less often unemployed and mothers more frequently had a higher level of education. These parents were also more likely to have attended a prenatal consultation with a neonatologist or visited a neonatal unit during pregnancy. In contrast, in the late SSC group, Caesarean section was more common, and infants were of lower gestational age. These infants more often had a birth weight below 1500 g and were more frequently admitted to a neonatal intensive care unit at birth.

**TABLE 1:** Baseline characteristics of the study population (2212 parents).

	Skin‐to‐skin contact within the first 3 days of life	Skin‐to‐skin contact day four onwards	*p* (chi‐square test)
	*n* = 987 (44.6%)	*n* = 1225 (55.4%)	
	Number (%)	Number (%)	
Questionnaire completed by the mother	948 (96.1%)	1168 (95.4%)	0.421
Mother's age^a^	30.8 (5.0)	30.7 (5.4)	0.438
Father's age^a^	32.1 (5.6)	31.9 (5.7)	0.260
Child's age when the parent completed the questionnaire^a^	2.6 (2.4)	3.6 (3.3)	< 0.001
Parent living alone at the time of the child's birth	30 (3.0%)	45 (3.7%)	0.413
Parent living alone when the questionnaire was completed	70 (7.1%)	108 (8.8%)	0.138
Mother went to university	705 (71.4%)	793 (64.7%)	0.001
Father went to university	502 (50.9%)	584 (47.7%)	0.200
Unemployed mother	25 (12.7%)	205 (16.7%)	0.008
Unemployed father	31 (3.1%)	60 (4.9%)	0.036
French is mother's native language	941 (95.3%)	1177 (96.1%)	0.390
French is father's native language	899 (91.1%)	1107 (90.4%)	0.657
Multiple pregnancy	178 (18.0%)	231 (18.9%)	0.620
Gestational age^a^	29.0 (1.8)	27.9 (2.0)	< 0.001
Birthweight less than 1500 g	815 (82.6%)	1135 (92.7%)	< 0.001
Child born before 2014	403 (40.8%)	701 (57.2%)	< 0.001
First pregnancy	700 (70.9%)	815 (66.5%)	0.027
Older sibling hospitalised at birth	58 (5.9%)	75 (6.1%)	0.556
Child deceased	88 (8.9%)	133 (10.9%)	0.130
High‐risk pregnancy	436 (44.2%)	497 (40.6%)	0.088
Hospitalisation during the pregnancy	609 (61.7%)	706 (57.6%)	0.053
Consultation with a neonatologist or visit to neonatal unit during the pregnancy	327 (33.1%)	377 (30.8%)	0.044
Delivery by Caesarean section	564 (57.1%)	775 (63.3%)	0.002
Delivery by Caesarean section under general anaesthesia	176 (17.8%)	331 (27.0%)	< 0.001
Admitted at birth to neonatal intensive care unit	555 (56.2%)	858 (70.0%)	< 0.001
Admitted at birth to local neonatal unit	403 (40.8%)	340 (27.8%)	< 0.001
Parents informed about the benefits of skin‐to‐skin care	946 (95.8%)	1161 (94.8%)	0.406

^a^ Mean (standard deviation), Mann–Whitney test.

**FIGURE 1:**
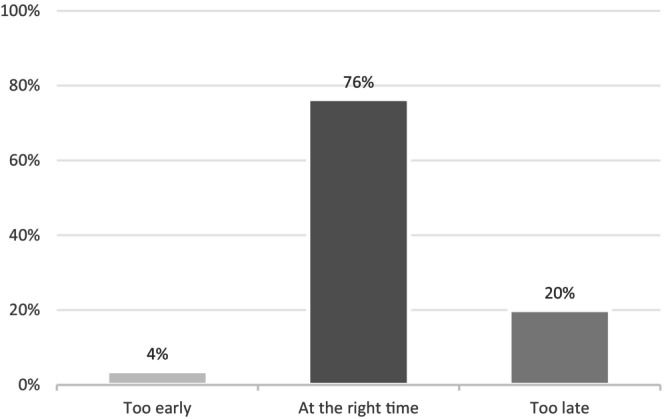
Parents' first skin‐to‐skin contact experience.

**FIGURE 2:**
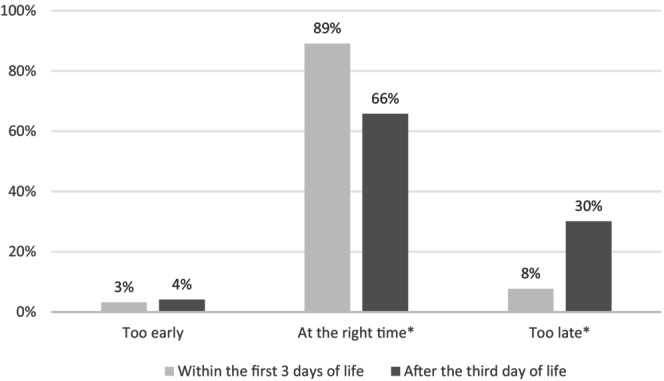
Parents' feelings according to the timing of the first skin‐to‐skin contact.

A small proportion of parents (4%) reported that the first SSC occurred too early (Figure 1). A statistically significant difference was observed between the two groups regarding perceptions of appropriate timing (Figure 2). In the early SSC group, the majority of parents (89%) considered the timing appropriate, compared with 66% in the late SSC group (*p* < 0.001) (Figure 2). Conversely, 8% of parents in the early SSC group felt that SSC occurred too late, compared with 30% in the late SSC group (*p* < 0.001). Similar results were found when analysing only responses from parents of infants born before 26 weeks' gestation.

**FIGURE 3:**
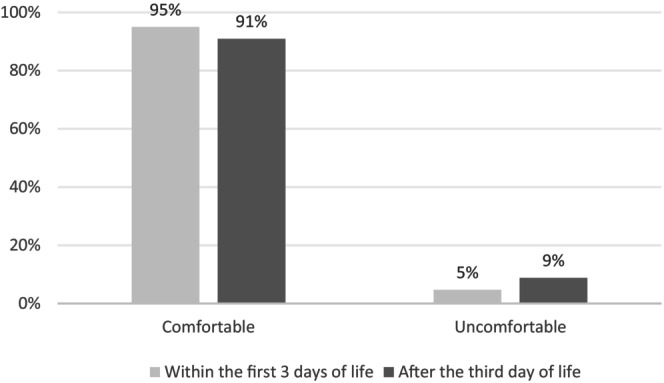
Parents' skin‐to‐skin contact experience during their infant's hospital stay according to the timing of the first skin‐to‐skin contact.

A greater proportion of parents in the early SSC group felt comfortable during SSC throughout the hospital stay (Figure 3): 95% versus 91% in the late SSC group (Figure 3, *p* = 0.001). After adjusting for gestational age, early SSC was significantly associated with increased parental comfort during the hospital stay (Table 2). The adjusted odds ratio was 1.64 (95% confidence interval 1.14–2.37, *p* = 0.008).

**TABLE 2:** Association between the timing of the first skin‐to‐skin care and parental feelings during skin‐to‐skin care throughout their infant's hospital stay.

	Bivariate model		Multivariate model	
	Unadjusted OR^a^	*p*	Adjusted OR^a^	*p*
Parents felt comfortable during skin‐to‐skin care	1.98 (1.38–2.82)	< 0.001	1.64 (1.14–2.37)	0.008

*Note:* Associated factor included in the final multivariate model: gestational age.

^a^ OR, odds ratios (95% CI), reference group = skin‐to‐skin contact after the third day of life.

Comments on the causes of stressful SSC were provided by 209 parents (9%). The most frequently reported cause of stress was fear (53%), fear of hurting the child and/or fear of doing wrong: ‘Fear of doing wrong! Or fear that something bad might happen to him during SSC and that I would not be able to help him’. ‘I always had this fear of hurting my child, of breaking him’. The baby's clinical condition, especially bradycardia/desaturation, was the second reason (37%) for a stressful SSC: ‘The sound of alarms in case of bradycardia and desaturation’. ‘If my son had oxygen desaturation during SSC, the nurses used to come to tell me that it was because I was not holding him well, that he was not feeling comfortable with me or that I was tiring him out’. Insufficient availability of staff during SSC was another cause (25%) of stress: ‘When my husband wasn't there, I needed someone to reassure me because my daughter could sense my stress and that caused dreadful desaturations’. ‘No bell to call someone and no nurse nearby’ Parents reported that the smallness and/or fragility of their baby was also a cause (21%) of stress: ‘My baby was so small’. ‘My lack of confidence came from my daughter's health and her frequent desaturations’. The lack of privacy during SSC was the last reported cause of stressful SSC (6%): ‘I did not feel there was enough privacy to be able to get undressed’.

A total of 427 parents (19% of the cohort) provided written suggestions to enhance the SSC experience. Many emphasised the importance of an appropriate environment (51%) and the availability of staff (25%) with comments such as: ‘Comfortable seating in an armchair, soft music and healthcare professionals nearby’. Other parents (16%) highlighted the need for their baby's clinical condition to be stable: ‘The baby's clinical condition should be stable enough’. Furthermore, some parents stressed the importance of respecting their mood and wishes (36%) concerning SSC, including being offered choices about the right time, the duration and frequency of SSC (14%). They also required explanations about the benefits of SSC for their baby and themselves (5%): ‘The main thing is that the parent should be ready and genuinely willing to practise SSC. A comfortable armchair is also important, and agreeing with healthcare professionals about the duration of SSC considering the baby's condition’. Another stated: ‘Healthcare professionals should explain the importance of SSC and help us feel comfortable’.

## 4 Discussion

This study, based on a large national online survey, found that the majority of parents of very preterm infants wished to initiate early SSC within the first 3 days of life. Furthermore, parents who practised early SSC were more likely to report feeling comfortable during their SSC time throughout their baby's hospital stay. Key conditions associated with a positive and non‐stressful SSC experience included an appropriate environment and sufficient privacy. Staff availability during SSC and the infant's perceived stable condition were considered essential. Respecting parental preferences and providing clear explanations about the benefits of SSC were also cited as key to a positive experience.

In France and Europe, SSC is now recommended for all hospitalised preterm infants, as early and as continuously as possible, as soon as clinical stability allows it [2, 24]. Our study showed that most parents of very preterm infants wish to practise SSC as soon as possible after birth. However, for most parents, the first SSC occurred after the third day of life. A Norwegian study published in 2023 looked at the impact of SSC on very preterm infants born vaginally and via Caesarean section [25]. Following a Caesarean section, the mother and the infant may be separated for several hours, or even days if the mother experiences complications. This can therefore delay the first SSC. In our population, 60% of births were by Caesarean section, 23% of which were under general anaesthesia. That is one reason why we decided to place the cut‐off for early SSC at 3 days in 2014. We chose to keep this cut‐off because it was also used in a French cohort study published in 2023 [26]. We realise that early SSC is now considered to be earlier, within the first day of life, and even that immediate SSC should be promoted, at least if the baby is not too premature [11, 15, 27, 28]. Facilitating SSC in the delivery and operating rooms required a redistribution of resources and the cooperation of more healthcare professionals than usual.

SSC improved in France from 2004 to 2011, according to a French cohort study published in 2016, especially in centres with formal early intervention programmes such as the Newborn Individualised Development Care and Assessment Programme [27]. However, as this study mainly involved parents of infants who were born after 2011, the results suggest that there remains room for further improvement. One way to promote early SSC could be to implement caring models, such as couplet care, which enable the infant and mother to receive care together from birth [29, 30].

This study showed that parental hesitation or discomfort existed. This must be acknowledged by healthcare professionals. According to the parents, this experience was due more to organisational factors such as a lack of professional support than to the infant's fragility, as this was found even for infants born before 26 weeks' gestation. To promote early SSC and parental comfort, parents require a clinically supportive environment during SSC, especially when their baby has frequent apnoea or bradycardia [13]. Written procedures facilitating infant transfer and monitoring during SSC, along with a supportive environment for both infant and parent, are essential [2]. Healthcare professionals might also invite parents to share their feelings and preferences, in order to adapt accordingly. In our survey, parents highlighted several ways to improve SSC during the hospital stay: a comfortable environment, responsive staff, clinical adaptation, and attention to the parent's health, mood and preferences.

The European Foundation for the Care of Newborn Infants has launched a transdisciplinary collaboration project to define standards for high‐quality perinatal and neonatal care [31]. Key standards relating to SSC include informing parents before birth about the importance of SSC and ensuring safe, early and continuous SSC involving both parents. A Swedish study published in 2022 analysed the interviews of 12 parents who experienced immediate SSC following the birth of their very preterm infants [16]. Parents reported that immediate SSC helped them recognise their roles as primary caregivers and fostered a calming physical connection with their newborn infant. Training healthcare professionals in safe SSC techniques is essential and up‐to‐date guidelines should be available in every neonatal unit. Ongoing administration of this parental survey will help monitor future developments and support continued improvements in SSC for very preterm infants. Parental online questionnaires are an effective tool for monitoring progress in neonatal care within the broader framework of infant and family‐centred developmental care. They should be used more regularly to track both practices and experiences, and improve care across neonatal units.

Hospital practices are often designed, created and implemented by expert healthcare professionals. For neonatal care, nowadays parents are involved as primary caregivers. Feedback about their experience is thus essential. In a context where SSC is being promoted, few papers have addressed parents' experience with SSC and their opinion on the timing of the first SSC. This research could help healthcare professionals implement SSC in a way that meets parental needs. Knowledge about the parental perspective may help implement an early, safe, supportive and comfortable SSC programme within each neonatal unit.

### 4.1 Strengths and Limitations

Our study focused on parents' feelings about their SSC experience in neonatal units in France and on the impact of early versus late SSC. There is little national data on parental experience. The other strength of this study is that it included a large number of parents who practised SSC with their baby who was hospitalised at birth.

According to the names of the hospitals and units mentioned by the parents, all neonatal units across France were represented.

Studies with open‐ended questions such as ours allow parents to express their feelings more precisely, in their own words.

One limitation of this study was the ability to generalise the results to populations without any internet or social media access, as participation required online access [22]. This study reflects mainly the mothers' perception. The prominent involvement of mothers in this survey likely reflects parental engagement realities in French neonatal units over that period. Changing this societal norm is beyond this study's scope, but is a concern in terms of parenting preterm infants. Nevertheless, since July 2020 in France, fathers of infants hospitalised at birth have been entitled to 30 days of paternity leave, added to the standard paternal leave. The standard paternal leave also increased from 11 to 25 days in July 2021. This remains less than in Nordic countries. The Nordic countries have been forerunners in parental leave since the 1970s [32]. France's policies align with most European Union member states. The recent policies aim to support fathers' involvement in the care of their neonate, as studies show fathers who take leave are more involved in childcare and housework [33, 34]. In this context, fathers in France may now be more involved in preterm care.

## 5 Conclusions

This study, based on a 10‐year online survey of parents, found that most parents of very and extreme preterm infants viewed early SSC as appropriate. SSC initiated within the first 3 days of life was associated with greater parental comfort with SSC during their infant's hospital stay. Parental needs should be anticipated and appropriately addressed to ensure a low‐stress, positive SSC experience.

## Funding

The authors have nothing to report.

## Conflicts of Interest

The authors declare no conflicts of interest.

## Data Availability

The datasets analysed during the current study are available from the corresponding author on reasonable request.
